# Population Health Could Do Far More to Mitigate Health Disparities

**DOI:** 10.1089/pop.2018.0042

**Published:** 2019-03-27

**Authors:** R. Scott Braithwaite

**Affiliations:** Department of Population Health, New York University School of Medicine, New York, New York.

**Keywords:** health disparities, patient autonomy, determinants

## Introduction

Population health – the health outcomes of a group of individuals, including the distribution of such outcomes within the group – aims to increase aggregate health and to mitigate health disparities.^1^ Despite, or because of, its broad scope, it is not illuminating a navigable path to mitigate health disparities. However, several areas of increased emphasis may amplify the field's effect: (1) address health-related research as a determinant of health disparities, (2) prioritize actionable programs, and (3) collaborate with bioethicists where goals may conflict with patient autonomy.

## Health-Related Research as a Determinant of Health Disparities

Health-related research is an underappreciated determinant of health disparities because it generates knowledge that fuels the “Fundamental Causes” theory, which stipulates that when new healthful knowledge arises those groups with higher socioeconomic status (SES) benefit more than groups with lower SES because of their greater material and nonmaterial resources.^2^ For example, the contribution of smoking to SES-related health disparities in 14 countries varied from 19% to 55% among men.^3^ This disparity would not exist if prior research had not been conducted demonstrating smoking's harms.

How might population health proactively address health disparities from differential adoption of healthful behaviors? Already, some approaches directly target behaviors in disparity-impacted groups, such as community outreach (eg, churches, barbershops) to address hypertension in low SES communities^4,5^ and elementary school-based programs that emphasize healthy eating and effective parenting in lower SES communities.^6,7^ However, a far wider range of approaches could be employed. Could substance harm reduction be linked to recent unemployment or to job retraining opportunities in industries buffeted by technological change? Could “dose-dependent” taxes be levied on unhealthful behaviors with dose-response effects (eg, sugar-sweetened beverages) by counterbalancing a general tax with an individual “shopper card”-like discount that gradually wanes as an unhealthy dose is approached? Although few or none of these approaches might be successful it is impossible to know without testing and evaluating them.

## Prioritize Actionable Programs

Many social risks (eg, income, education, SES) are tightly interwoven with unhealthy behaviors and with poorer health outcomes. Approximately one half of their effects are mediated through unhealthy behaviors.^2^ The remaining half is mostly uncharacterized, but many potential explanations lie beyond the reach of available data. For example, population-level data that include potentially important psychological factors such as impulsivity, self-efficacy, locus of control, conscientiousness (the propensity to be goal-directed, responsible, and in control of impulses), and neuroticism (frequent experience of negative emotions and emotional instability) rarely analyze them in conjunction with social risks or unhealthy behaviors. Indeed, these last 2 constructs may have relationships with mortality that are as robust as social risk factors,^8,9^ although they have not yet been examined concurrently with social risks and unhealthy behaviors to assess their relative contributions. Consequently, there likely are uncharacterized but important synergies between individual psychological factors, culturally normative behaviors, and unhealthy behaviors.

Although it is important to gather more information in the future, we can presently emphasize known contributors that are most actionable. In particular, some unhealthful behaviors may be “low-hanging fruit,” changeable without altering social contexts, entrenched behaviors, or individual psychological factors. For example, a randomized controlled trial of school-based parental training shows improved childhood development and lowered obesity,^6^ and increasing the Earned Income Tax Credit appears to improve child development.^10^

Identifying actionable approaches in population health is especially challenging because the relationship of social risk factors to health outcomes has been spared the scrutiny given to the relationships of non–social risk factors. Although Braveman et al^11^ suggest investigators should “carefully trace the series of causal inferences from intervention to effect; at each step, relevant evidence should be documented and distinctions between associations and likely causal links should be carefully considered,” this type of rigorous inferential thinking is often absent.^12^ For example, peer-reviewed publications have suggested that approximately 245,000 US deaths in 2000 were attributable to low education, 176,000 to racial segregation, 162,000 to low social support, 133,000 to individual-level poverty, 119,000 to income inequality, and 39,000 to area-level poverty.^13^ Such tenuous inferences amplify the linguistic defect in epidemiological terminology, where “population attributable risks” and “population attributable fractions” employ the language of causality without bearing its meaning.^14^ Further, tenuous inferences are mistakes for 2 reasons: First, they forgo the opportunity to target resources to more effectively diminish health disparities (eg, would we maximally reduce health disparities by investing resources in more green areas, safe walking spaces, availability of fresh produce, recreational sites for physical activity, or personal safety from crime?). Second, they motivate premature closure on the forms assumed by “upstream” determinants of health, including factors that are underexamined because of data limitations (eg, psychological attributes). Explicitly considering the likelihood of causality combined with potential for change could help focus limited resources on those interventions with the best prospects for reducing disparities.

## Ensure That Health Disparity Mitigation Does Not Impinge on Autonomy

Health disparities may be manifestations of preferences or autonomy if they are not health inequities (eg, not linked to unjust or discriminatory burdens). Efforts to discern this distinction are important in order to preserve individual autonomy, and to ensure preference-sensitive and culturally-competent health care and policies.

For example, defibrillators are implanted less frequently into blacks than whites with comparable clinical indications.^15^ But is this because black patients receive counseling less often than white patients or because black patients do receive counseling but are less likely to elect surgery? These explanations have very different implications. Being less likely to receive counseling is an inequity and a disparity in care. However, being less likely to elect surgery could reflect a preference for less invasive care that is not necessarily an inequity or disparity in care.^16^ Targeting comparable defibrillator implantation rates in black and white patients could override patient autonomy and could contradict preference-sensitive care. Similar questions apply to joint replacement rates disparities^17^ and other preference-sensitive decisions. A target body mass index (BMI) of 28 rather than 25 is a preference-sensitive decision because different persons and cultures attach different non–health valuations to moderate levels of overweight that have minor health implications. Therefore, differences in attainment of BMI ≤25 may represent a disparity, but a desirable one, because its elimination would contradict the ethos of respecting personal and cultural preferences.

## Conclusion

The field of population health has great potential to mitigate health disparities. However, this potential may be unrealized without greater awareness of health research as a contextual determinant of health disparities, emphasis on actionable goals, and evaluation of bioethical implications.
